# Control of adult neurogenesis by programmed cell death in the mammalian brain

**DOI:** 10.1186/s13041-016-0224-4

**Published:** 2016-04-21

**Authors:** Jae Ryun Ryu, Caroline Jeeyeon Hong, Joo Yeon Kim, Eun-Kyoung Kim, Woong Sun, Seong-Woon Yu

**Affiliations:** Department of Anatomy, College of Medicine, Korea University, Seoul, 136-705 Republic of Korea; Department of Brain and Cognitive Sciences, Daegu Gyeongbuk Institute of Science and Technology (DGIST), Daegu, 711-873 Republic of Korea; Neurometabolomics Research Center, Daegu Gyeongbuk Institute of Science and Technology (DGIST), Daegu, 711-873 Republic of Korea

**Keywords:** Adult neurogenesis, Programmed cell death, Neural stem cells, Neuroblasts, Apoptosis, Autophagy, Necrosis

## Abstract

The presence of neural stem cells (NSCs) and the production of new neurons in the adult brain have received great attention from scientists and the public because of implications to brain plasticity and their potential use for treating currently incurable brain diseases. Adult neurogenesis is controlled at multiple levels, including proliferation, differentiation, migration, and programmed cell death (PCD). Among these, PCD is the last and most prominent process for regulating the final number of mature neurons integrated into neural circuits. PCD can be classified into apoptosis, necrosis, and autophagic cell death and emerging evidence suggests that all three may be important modes of cell death in neural stem/progenitor cells. However, the molecular mechanisms that regulate PCD and thereby impact the intricate balance between self-renewal, proliferation, and differentiation during adult neurogenesis are not well understood. In this comprehensive review, we focus on the extent, mechanism, and biological significance of PCD for the control of adult neurogenesis in the mammalian brain. The role of intrinsic and extrinsic factors in the regulation of PCD at the molecular and systems levels is also discussed. Adult neurogenesis is a dynamic process, and the signals for differentiation, proliferation, and death of neural progenitor/stem cells are closely interrelated. A better understanding of how adult neurogenesis is influenced by PCD will help lead to important insights relevant to brain health and diseases.

## Background

Programmed cell death (PCD) is a type of cell death by design that mainly occurs during embryonic development. The presence of neuronal PCD during development was first discovered by Beard in the 19th century, and the extent of PCD in many different neuronal populations, the underlying molecular mechanisms, and the biological roles of PCD have been studied since the 1980’s [1]. In particular, discoveries of key molecules regulating PCD, including caspases and the Bcl-2 family proteins, have fueled the study of developmental PCD. The biological importance of PCD has also been addressed using animals with mutations in genes controlling PCD. The roles of PCD during nervous system development appear to be classified into three main categories: 1) regulation of the size of progenitor populations, 2) error correction and 3) systems matching. Each of these roles is associated with the optimization of systems by the removal of erroneous or unwanted neural populations for sculpting the nervous system during development. One of the most studied types of neuronal PCD is the target-dependent survival of peripheral neurons. For example, about 50 % of sensory neurons or motor neurons innervating their targets (e.g. skin or muscle, respectively) eventually undergo PCD during their provisional synapse formation. Because supplements of additional targets or appropriate neurotrophic factors rescue them from death, it is believed that target-derived neurotrophic signals are responsible for the survival of innervating neurons (neurotrophic hypothesis). Competition among innervating young neurons is critical for the selection of surviving vs. dying neurons, and the limited amount of neurotrophic factors appears to determine the extent of PCD [2–7]. However, how neurotrophic factors select ‘winners’ and ‘losers’ are less understood.

More recently, the presence of neural stem cells (NSCs) and the spontaneous production of new neurons from these cells in the adult brain have been identified. Adult NSCs are cells that have the potential to self-renew and differentiate into multiple cell types including neurons, astrocytes, and oligodendrocytes in the adult nervous system [8]. Newly generated neurons derived from adult NSCs become physiologically mature and are functionally integrated into the preexisting neural circuit [9]. They are known to be involved in several brain functions and behaviors including learning and memory [10, 11]. Adult neurogenesis is a dynamic process recapitulating the development of the embryonic nervous system and is modulated by various physiological and pathological activities at all stages: proliferation and differentiation of NSCs, migration, survival, maturation, and integration of newborn neurons.

Because adult neurogenesis is an extension of the embryonic development of the nervous system, PCD also plays an important role in the regulation of adult neurogenesis and the integration of young neurons into mature neural circuits [12]. Although the molecular machineries controlling PCD appear to be the same in the developing and adult brains, neuronal competition in particular is quite different. In the developing nervous system, competition for neuronal survival occurs among immature embryonic neurons with similar developmental/differentiation stages. On the other hand, young neurons in the adult brain likely compete with young neurons in similar stages and with preexisting mature neurons for survival and/or circuit integration. In this respect, PCD in the adult brain may have unique regulatory mechanisms or biological utility; only a few studies have addressed this issue. In this review, we focus on current knowledge of adult neurogenesis and the contribution of PCD as a regulatory strategy, and discuss potential roles of PCD in adult brain function.

## Neurogenesis and PCD in the adult brain

Neurogenesis was traditionally accepted to occur during embryonic developmental in the mammalian central nervous system (CNS), but in the 1960s it was suggested by Altman and Das that new neurons from progenitor cells were continuously added throughout adulthood [13]. Since then, technical advances have allowed researchers to demonstrate adult neurogenesis. The identification of newborn neurons in the adult CNS has been made possible in large part by three different approaches: 1) analyzing the incorporation of the nucleotide analogues [^3^H]-thymidine or bromodeoxyuridine (BrdU) during cell division, 2) genetic marking by stereotaxic injection of retrovirus carrying a reporter gene into the adult brain, and 3) tracking the expression of specific markers and the development of transgenic models under specific promoters [14]. Although these approaches have been used successfully to investigate adult neurogenesis, they each have disadvantages and limitations, leading to discrepancies among studies. Although nucleotide analogues can be used to cover the entire population of newborn neurons, their incorporation does not occur only during cell division. They can be incorporated into newly synthesized DNA resulting from the DNA repair process and even in postmitotic neurons after brain injury [15]. Furthermore, these nucleotide analogues may influence the normal process of adult neurogenesis [16]. Retroviral injection is useful for the direct visualization of newborn neurons, but it requires invasive surgical procedures and covers only a limited number of new neurons [17–21]. Non-invasive genetic labeling of adult-born cells in transgenic animal models, such as the inducible Cre recombinase and tamoxifen-regulated system (Cre-ER), is an alternative approach that is now being successfully applied [22–25]. Specific markers for NSCs and newborn neurons cover the entire population of newborn cells and can be used for human samples, but they are expressed transiently and sometimes nonspecifically in other cell types. Therefore, these approaches should be used carefully, and a combination of these methods will help avoid false interpretation of the results. Recently, a birth-dating method to measure carbon-14 (C^14^), derived from nuclear bomb testing, has been applied to demonstrate adult neurogenesis in humans [26–28].

Although various techniques have been developed to easily detect the generation of new neurons in the adult brain, the assessment of PCD in the adult brain is not easy. Dead cells are efficiently removed by immune cells such as microglia in the brain [29, 30]. In some cases, immature neuroblasts also contribute to the clearance of dead cells in the dentate gyrus [31]. Therefore, the presence of dead cells may be transient as a result of active clearance and may render the identification and quantification of dead cells in the adult brain very complicated [32]. In this respect, knockout mice that fail to execute PCD, such as Bax-knockout mice, have been efficiently used to assess the extent and distribution of PCD in the adult brain [33, 34].

**Fig. 1:**
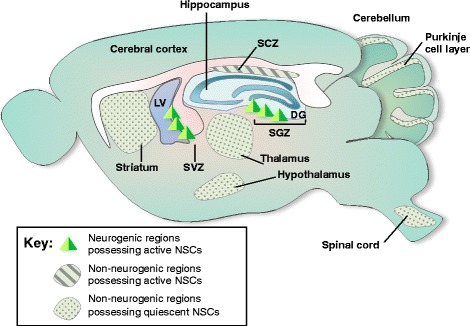
Adult neurogenesis in spontaneous neurogenic regions and non-neurogenic regions. *Neurogenic regions possessing active neural stem cells (NSCs):* In the adult brain, neurogenesis in the subgranular zone (SGZ) of the hippocampal dentate gyrus (DG) and the subventricular zone (SVZ) of the lateral ventricle actively supplies newly generated cells. SGZ and SVZ have been identified as spontaneous neurogenic regions possessing self-renewing neural stem cells (NSCs) and neural progenitor cells (NPCs), respectively. *Non-neurogenic regions possessing active NSCs:* In addition to these two discrete regions, subcallosal zone (SCZ) is the sources for continuously generating multi-potent NSCs. *Non-neurogenic regions possessing quiescent NSCs:* Recent reports have suggested that NSCs may be widely distributed in the adult brain. The existence of NSCs is proposed by in vitro neurosphere culture and BrdU^+^ labeling in many regions which were previously believed to be non-neurogenic, such as striatum, thalamus, hypothalamus, spinal cord, and Purkinje cell layer of the cerebellum. One of the difficulties for identifying NSCs in the non-neurogenic regions is possibly due to the mitotic quiescence of the NSCs, which has inducible capacity for self-renewal and multi-potency under pathological conditions

Adult neurogenesis actively occurs in spatially restricted brain regions of the mammalian CNS throughout adulthood [35]. Spontaneous neurogenic regions refer to brain regions that spontaneously produce new neurons in adults under normal conditions. The subventricular zone (SVZ) of the lateral ventricle and the subgranular zone (SGZ) of the hippocampal DG in adults continuously and spontaneously generate neurons under normal conditions. Although adult neurogenesis in these two spontaneous neurogenic regions is well established, reports of the presence of NSCs and spontaneous adult neurogenesis in other brain regions, called non-neurogenic regions, are conflicting (Fig. 1). Neurosphere assays and the labeling of specific markers have identified the presence of NSCs in the spinal cord, cerebral cortex, cerebellum, and retina [36–39].

### PCD in neurogenic regions possessing active NSCs

#### SVZ

NSCs in the adult SVZ, located adjacent to the ependymal cell layer of lateral ventricles, proliferate and differentiate to immature neurons. Newborn neurons in this region migrate tangentially into the olfactory bulb (OB) through the rostral migratory stream to become granule neurons and periglomerular neurons [40]. The RMS is guided through chain migration via the formation of elongated cell aggregates. During migration, blood vessels are closely associated with chains of cells to form a scaffold for migration [41, 42]. At 2 weeks after birth in the adult brain, most newborn neurons have reached the OB and move radially toward the granule cell layer and the periglomerular cell layer in the OB. This migration is regulated by interactions between cells or between the cell and the extracellular matrix; the ephrin family of proteins, ErbB4, neural cell adhesion molecule (NCAM), and reelin are known to be involved in this process [43]. Secretory signals, such as hepatocyte growth factor (HGF), glutamate, and gamma aminobutyric acid (GABA) also contribute to the regulation of chain migration [44–46]. Newborn neurons become more complex in morphology, developing elaborate dendrites and axon. Granule neurons are morphologically mature at 2 weeks and periglomerular neurons at 4 weeks after their birth. During maturation, they form synapses, receiving synaptic inputs through dendritic spines. It has been estimated that 60,000–120,000 cells in 2-month-old rats and 30,000 cells in adult mice are integrated into OB neural circuits daily [33, 47–49]. However, 50 % of neural progenitor cells (NPCs) and young neurons undergo PCD to eliminate superfluous cells, and the remaining neurons can survive up to 1 year [49, 50]. Neurogenesis in the SVZ is regulated by diverse mechanisms. Sensory input has been shown to be critical for the survival of adult-born neurons during neuronal maturation [50]. Neurotrophic factors [51, 52], growth hormones [53], and neuropeptide Y [54, 55] have been reported to play a role in adult SVZ neurogenesis. Although the function of adult SVZ neurogenesis is unclear, it is involved in odor memory [10, 56–58] and pheromone-related social interactions [59].

It is important to note that the SVZ is poorly developed in humans, and compared with the rodent SVZ, the human SVZ has lower numbers of proliferating cells and neurons [60–62]. Arguably, the human brain lacks chains of migrating neuroblasts from the SVZ to the OB [62–64]. At this point, it remains unclear whether this structural difference requires different regulatory mechanisms in humans and rodents.

#### SGZ

In the adult SGZ, hippocampal NSCs are juxtaposed with a dense layer of granule cells in the DG. They proliferate and differentiate into immature granule neurons. Depending on the context, 30–70 % of the newborn neurons survive, but the remainder die between the first and second week after birth. Surviving neurons then migrate into the granular cell layer and send axons to form functional synapses on CA3 pyramidal neurons 2 weeks after birth, and the newly formed synapse becomes stable at 4 weeks [65]. Dendrites from the newborn neurons reach the outer molecular layer within 4 weeks [66]. During maturation, dendritic refinement and synapse formation in newborn neurons are very tightly regulated by diverse mechanisms. Silencing of Disrupted-In-Schizophrenia-1 [67], class 3 Semaphorin [21], kinesin II motor protein [68], and glucocorticoid receptors [69] in newborn granular neurons affects their dendritic growth and cell positioning. In addition, aging, electrical activity, enriched environment, and physical exercise dynamically regulate the proliferation of NSCs and the survival and differentiation of newborn neurons in the adult hippocampus [14, 70, 71]. Approximately 30–70 % of newly generated neuroblasts are eliminated within the first month after birth during their integration period, depending on the animals’ physiological/pathological condition and their experience [72]. We have estimated how many neurons undergo PCD by analyzing the number of newborn neurons in Bax-knockout mice in which PCD is completely blocked [73]. The number of DG cells in Bax-knockout mice increased 2-fold by 12 months compared to WT mice, whereas DG cell numbers were similar at 2 months. These data suggest that PCD is marginal during the early postnatal period when the DG is forming, but PCD is an important factor for regulating adult neurogenesis. It appears that 1,300 cells undergo PCD daily and are replaced by newborn neurons in the adult mouse DG. Comparably, in humans, 700 new neurons, or 0.004 % of DG neurons, are added in each hippocampus per day [74]. This means that 1.75 % of the neurons within renewing populations are annually renewed in the human brain during adulthood. These results indicate that PCD also plays a significant role in the regulation of adult neurogenesis in humans.

### PCD in non-neurogenic regions possessing active NSCs

#### SCZ

Recently, the subcallosal zone (SCZ) has been identified as a neuroblastogenic region in the adult brain [75]. The SCZ is located beneath the neocortex and is closely associated with white matter. Most cells born in adult SCZ migrate into the corpus callosum and become oligodendrocytes [37]. Compared with the SVZ and SGZ, newborn neurons in the SCZ undergo virtually complete PCD regulated by Bax. Newborn neurons survive in Bax-knockout adult mice but not in wild-type mice; however, they fail to become functionally mature neurons due mostly to the absence of appropriate neurotrophic supports [38, 75]. A functional role for neurogenesis in the adult SCZ is indicated by the fact that focal traumatic brain injury promotes neurogenesis in this region, which suggests that the adult SCZ maintains neurogenic potential that could contribute to recovery in the injured brain. Although the biological utility of neuroblastogenesis in the SCZ is yet unclear, this observation suggests that PCD is one of main strategies involved in determining the content of neurons integrating into mature neural circuits. Similarly, dividing Pax6- and Olig2-positive NSCs within the subcortical white matter continually produce doublecortin (DCX)-positive new neurons, but they are lost within a week of their birth [38].

The SCZ, which, in the human brain, is often called subcortical white matter, is well-developed in humans, and the embryonic SCZ contains transient neurons that might be postnatally eliminated by PCD [76]. Previously, Roy et al. found that the human SCZ possess mitotically competent progenitor cells, most of which differentiate into oligodendrocytes in vitro*;* however, a very small fraction of these cells are found to mature into neurons [77]. These observations indicate that these progenitors have the potential for multilineage differentiation. Later, this group of researchers confirmed that human white-matter progenitor cells from the SCZ can produce functionally mature neurons and glia in vitro and after xenograft to the fetal rat brain [78]. However, further studies are needed to understand the regulatory mechanism of adult neurogenesis in the human SCZ, including PCD.

### PCD in non-neurogenic regions possessing quiescent NSCs

The isolation of proliferating cells from many non-neurogenic regions in the adult brain and their differentiation into neurons in vitro and in vivo after transplantation has led to the emerging view that NSCs may be widely distributed in the adult brain, although there are still discrepancies among studies. Whether neurogenesis occurs in the adult neocortex is heavily debated. Several reports have shown that the neocortex generates neurons in the adult rat [79], hamster [80], and macaque [81, 82], whereas others have shown that there are no adult-born neurons in the neocortex [27, 83, 84]. Which regions of the brain produce newborn neurons in the neocortex are also matters of debate. It has been shown that new neurons are added to the neocortical association areas in the adult macaque, but it appears that they migrate from the SVZ through the white matter [81]. The fact that NG2-immunoreactive neurons were found in the neocortex indicates newborn neurons originate from the cortex itself, as neural/glial antigen 2 (NG2)-positive precursors reside within this area [79]. In fact, many recent studies have reported that new neurons are generated in the adult mammalian neocortex under pathological conditions [46, 85, 86]. These reports suggest the presence of NSCs in the neocortex, which can be upregulated by brain injury.

Contradictory results, however, have been shown in other brain regions including the striatum, amygdala, substantia nigra, brainstem, olfactory tubercle, and piriform cortex [87]. The neurogenic potential of the adult spinal cord was identified by characterizing neurosphere-forming cells isolated from the adult spinal cord [64, 88]. The number of putative NSCs from the adult spinal cord is greatly increased by spinal cord injuries [89, 90]. The nature of these neurosphere-forming cells in terms of whether they possess ependymal or glial characteristics is still under debate [91–94].

Growing evidence suggests that the adult cerebellum contains cells with characteristics of NSCs. Bergmann glia, radial glia present in the Purkinje cell layer, express the neural stem markers Sox1/2/9 in the adult cerebellum [74, 95]. Purified NSCs from the postnatal murine cerebellum can form self-renewing neurospheres and differentiate into multiple cell types including neurons in vitro and in vivo after transplantation [96]. Upon transplantation to the perinatal cerebellum, NSCs from the cerebellum differentiate into region-specific cerebellar neurons and acquire the mature electrophysiological properties of cerebellar granule cells [97].

A small group of retinal stem cells persists at the margin of the retina in most vertebrates. Retinal stem cells in fish and amphibians continue to produce progenitors throughout life, adding new retina to the periphery of the existing retina. This new retinal addition is limited in birds and not evident in mammals. Putative NSCs from the retinal periphery and ciliary body of mammals can be isolated and grown in vitro for extended periods [36]. A variety of cell types have been suggested to potentially act as neural progenitors in the adult mammalian retina, including Müller glia [98], ciliary epithelium [64], and iris pigment epithelial cells [76]. The neurogenic potential of Müller cells was initially identified in lower vertebrates [63, 99] and later in humans [100]. Ciliary epithelial stem cells are present in a mitotically quiescent state in the adult mammalian eye and under in vitro conditions, they begin to express sodium and potassium channels and thereby develop into functional neurons [64]. They also respond to specific cues in culture conditions and preferentially differentiate along the lineages of retinal ganglion cells (RGCs) and rod photoreceptors [101]. Similar to other retina stem cells, pure isolated iris pigment epithelial cells express neural progenitor markers and differentiate into neurons and glial cells [76].

Adult neurogenesis in the hypothalamus has also been reported [102, 103]. Hypothalamic radial glia-like ependymal cells (tanycytes) from the median eminence generate newborn neurons in the adult hypothalamus [104]. Neurogenesis in the adult hypothalamus is involved in determining the weight and metabolic activity of adult mice [102]. Furthermore, postnatal hypothalamic neurogenesis is regulated by a high-fat diet [105]. In addition to the hypothalamus, the striatum, septum, and thalamus maintain NSCs that are activated upon exposure to certain growth factors and regulatory signals *in situ* [102, 106]. Additional studies have also reported on the production of new neurons in the rat midbrain [107], although this remains controversial [108, 109].

Notably, an in vitro neurosphere assay has been used to demonstrate the presence of NSCs in many non-neurogenic regions by assessing the potential of distinct populations to form neurospheres in vitro. However, a recent publication by Codega et al. challenges the utility of this assay to identify ‘quiescent’ stem cells in vivo. Quiescent NSCs, which rarely form neurospheres or adherent colonies in vitro*,* might be present in distinct brain regions in vivo. Only activated NSCs, which actively divide in vivo*,* can form neurospheres in vitro [110]. This suggests that there might be additional non-neurogenic regions that possess potential NSCs, possibly quiescent NSCs, which are not actively dividing and neurosphere-forming NSCs.

In summary, accumulating evidence has shown that adult NSCs are present in many brain regions other than the spontaneously neurogenic SVZ and SGZ, although they cannot be easily identified because of their mitotic quiescence, slow dividing activity, or potentially massive PCD under normal conditions (Fig. 1). Therefore, it becomes more important to explore the mechanism by which non-neurogenic brain regions regulate the proliferation of NSCs and their differentiation into neurons in normal and pathological conditions in order to better understand adult neurogenesis.

## Principles and key molecules of PCD

**Fig. 2:**
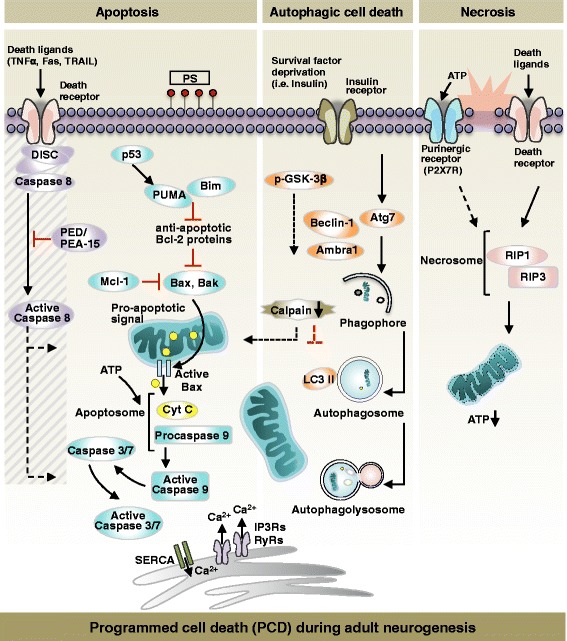
The key players in the regulation of PCD during adult neurogenesis. *Apoptosis:* Pro-apoptotic signals induce Bax translocation to the outer membrane of mitochondria. Bax-mediated pore formation leads to the release of apoptogenic cytochrome c (Cyt c) into the cytosol and apoptosome formation. The apoptosome activates procaspase-9 and catalytically active caspase-9 induces activation of downstream effector caspases, caspase-3 and -7 (Caspase 3/7). Phosphatidylserine (PS) exposure at the cell surface is required for the clearance of apoptotic cells. In the adult neural stem cells (NSCs), p53, Bim and PUMA have been implicated in activating apoptosis. In addition, the pro-apoptotic proteins, Bax and Bak are the key regulators. Mcl-1 antagonizes the pro-apoptotic proteins, and therefore, considered as a critical anti-apoptotic protein for the survival of the NSCs. In extrinsic apoptosis, a death ligand (TNFα, Fas, or TRAIL) binding activates death receptor and induces DISC complex formation near the receptor. Upon DISC complex-mediated activation of caspase-8, intrinsic and extrinsic apoptosis converge at the level of the executioner caspase cascade. In adult NSCs, PED/PEA-15 represses the activation of caspase-8. Release of Ca^2+^ from the ER and subsequent transfer to the mitochondria promtes the commitment of NSCs to cell death. *Autophagic cell death (ACD):* Autophagy is induced when cells are starved of nutrients or survival factors. Atg7 regulates the maturation of autophagosome and initiates the lipidation of LC3 (also called LC3 II). Cargos subjected to degradation are degraded in the autophagolysosome. AMBRA1 and Beclin-1-induced autophagy is inversely correlated with apoptosis in adult NSCs. Under insulin-deprived condition, the adult hippocampal neural stem (HCN) cells succumb to ACD wherein the cell fate is under the control of GSK-3β activation. Inhibition of GSK-3β phosphorylation (p-GSK-3β) induces ACD. The negative regulator of ACD is calpain, which also mediates the crosstalk between apoptosis and ACD. *Necrosis:* Extracellular ATP or death receptor activation rapidly induces RIP1/RIP3 necrosome formation. Necrotic cell death results from the depletion of cytoplasmic ATP due to mitochondrial dysfunction. A purinergic P2X7 receptor-mediated necrosis induction has been reported in adult NSCs. However, regulator of necrosis in adult NSCs has not been identified to date

The molecular process and causality of PCD in the CNS have been central topics of continuous research in the past decades. We briefly highlight core regulatory molecules and biochemical pathways of apoptosis, autophagy, and necrosis in this section, followed by a review of new insights into PCD for the control of adult neurogenesis in the next section (Fig. 2).

### Apoptosis

Cells undergoing apoptosis can be morphologically distinguished by the progressive appearances of the following features: 1) cytoplasmic condensation, 2) nuclear shrinkage (pyknosis), 3) nuclear fragmentation (karyorrhexis), and 4) non-immunogenic engulfment by nearby macrophages (phagocytosis) [111, 112].

Apoptosis is categorized into two types: intrinsic and extrinsic. In the mitochondrial pathway of intrinsic apoptosis, Bax and Bak activation causes: 1) permeabilization of the mitochondrial outer membrane, 2) the release of mitochondrial apoptogenic proteins including cytochrome c to the cytosol through protein permeable-pores and 3) the execution pathway involving the activation of caspases (mainly caspase-3, caspase-6, and caspase-7) [113–115]. Lastly, apoptotic cells expressing phosphatidylserine at the external surface are eliminated by phagocytic cells [116]. Overexpression of the pro-survival Bcl-2 family proteins, i.e.,Bcl-X_L,_ prevents Bax oligomerization in the outer membrane of the mitochondria, protecting cells from commitment to the apoptotic pathway [117, 118]. Several death effectors, including DNA damage, induce caspase-independent apoptosis via the translocation of AIF1 from mitochondria to the nucleus [119].

Extrinsic apoptosis is initiated by ligand-mediated activation of death receptors, including those for tumor necrosis factor (TNF), Fas, TNF-related apoptosis-inducing ligand (TRAIL), etc. [120]. Ligand binding stimulates the assembly of the death-inducing signaling complex (DISC)—which includes FADD and pro-caspase-8—near the receptor [120]. After caspase-8 activation, the extrinsic apoptosis pathway converges with the intrinsic apoptosis pathway at the level of executioner caspases [116].

### Autophagy

Autophagy (from Greek, meaning ‘self-eating’) is a catabolic pathway by which bulk cytoplasmic materials—including macromolecules and organelles—are degraded in a selective or a non-selective manner [121]. Cells undergoing autophagy display autophagic vacuolar structures including autophagosomes and autolysosomes, the products of autophagosome fusion with lysosomes [122]. The process of synthesis of autophagosome, the engulfment of autophagy substrates, the autophagolysosomal maturation, and the degradation of materials by lysosomal hydrolases is denoted as ‘autophagy flux’ [123]. Through its role in recycling proteins and catalyzing dysfunctional cytoplasmic organelles, autophagy is undoubtedly involved in a variety of physiological cellular processes including cell survival and death, metabolism, development, and aging.

Besides its role in maintaining cellular homeostasis, autophagy is a direct contributor to cell death in a variety of species including yeast, *Drosophila*, and mammalian cells [124]. Although apoptosis, autophagy, and necrosis have distinct signaling pathways, many biochemical components converge. Therefore, apparent changes in the expression of autophagy-related molecules are insufficient to implicate autophagy as a direct contributor of cell death. Based on accumulating evidence for the role of autophagy in PCD, recent studies have established discernible criteria for autophagic cell death (ACD): 1) cell death does not accompany the activation of apoptosis executers; 2) dying cells have increased autophagic flux; and 3) autophagy suppression rescues or prevents cell death [125, 126].

### Necrosis

The death ligands that drive cells to apoptosis can kill cells via necrosis as well [127]. Although necrosis is one of the three modalities of PCD, necrosis-specific molecular processes leading to cell death in a programmed manner are relatively unknown. Receptor-interacting protein (RIP) kinases are core regulators of necrotic cell death [128]. Knockdown or chemical inhibition of RIP1 by necrostatin-1 inhibits TNF-induced necrotic cell death [128].

The intracellular concentration of ATP provided either by glycolysis or mitochondrial respiration is a determinant of the molecular switch between apoptosis and necrosis; prolonged depletion of intracellular ATP levels shifts cells to undergo necrotic cell death [129, 130].

## PCD for the control of adult neurogenesis

Aiming to understand factors that control the balance between NSC proliferation and death during neurogenesis in the adult brain, this section will attempt to bring together existing in vivo evidence supporting the idea that adult neurogenesis is regulated by PCD with in vitro molecular studies to gain insight into the molecular and cellular mechanisms of PCD during adult neurogenesis. It is very challenging to directly demonstrate the molecular mechanisms of cell death of NSCs in vivo; therefore, it is imperative to extrapolate the relevant mechanisms and potential roles of cell death regulators in neurogenesis from in vitro biochemical studies [131]. Therefore, we will mostly consider studies of NSC death in in vitro NSC cultures in order to identify mechanisms that could provide insight into adult neurogenesis in vivo, because our particular interest is in understanding the molecular mechanisms of PCD and how it affects neurogenesis in the adult brain.

### Apoptosis of NSCs

#### Intrinsic apoptosis in the adult NSCs

The survival of NSCs is dependent on growth factors in neurogenic niches. FGF-2, a mitogen, is one of the factors that regulate proliferation and postmitotic cell fate [132]. When deprived of growth factors, NSCs undergo apoptosis. Adult NSCs possess multiple checkpoints along the PCD signaling pathway to repress or promote cell death. For example, NSCs from *bax*^−/−^*bak*^−/−^ mice are resistant to growth factor deprivation-induced apoptotic cell death. Also, adult NPCs in the SVZ are more susceptible to apoptosis when Mcl-1 is conditionally knocked out, whereas Mcl-1 overexpression reduces endogenous apoptosis by 50 % in nestin-positive adult NPCs from the SVZ [133]. These reports indicate that mitochondrial apoptotic pathway is essential PCD in newly generated cells in the neurogenic regions [73] and Bcl-2 family proteins function as regulators of NSCs commitment to mitochondria-dependent apoptosis. As such, bax-deficient NSCs are viable when subjected to apoptosis-inducing stimuli such as staurosporine, a prototypical inducer of apoptosis which inhibits multiple kinases, or etoposide [134]. On the other hand, adult wild-type NSCs show characteristic mitochondrial apoptotic markers, including the release of cytochrome c, cleaved caspase-3, and DNA fragmentation when stimulated with staurosporine [135]. Although mitochondria-dependent apoptotic pathways seem to be critical in the death of NSCs derived from different neurogenic regions, further comparative studies are required to understand regional differences in the molecular signaling mechanisms and reveal a unified or region-specific mechanism of the intrinsic apoptosis in the adult NSCs.

In the DG, Bim or PUMA deficiencies also lead to significant enhancement of the survival of newly developed neurons [136]. PUMA is a downstream target gene of p53, which is a transcriptional regulator of apoptosis upon cellular stress [137]. p53-dependent apoptosis in NSCs of DG and OB is mediated by PUMA [137, 138]. In addition, p53 deficiency enhances proliferation and induces differentiation toward neuronal and glial cell types in SVZ [139]. However, in contrast to these reports on the inhibition of NSC self-renewal by p53, there are reports on the surprising increase of cell death in the neurogenic regions of mice deleted of p53 [140, 141]. Therefore, the exact role of p53 in regulation of NSCs remains unsettled.

Recently, a number of studies have shown that endoplasmic reticulum (ER)- mitochondrial Ca^2+^ promotes the commitment of NSCs to cell death. The close proximity of the ER and mitochondria permits synergistic intracellular signaling events, including lipid biosynthesis, Ca^2+^ transmission, and mitochondrial fusion and fission dynamics [142]. Ca^2+^ transfer at the ER-mitochondria contact site mediates the initiation of apoptosis. VDAC1, Akt, Bax, Bak, and Bcl-2 modulate the discharge of intracellular Ca^2+^ from the ER to mitochondria via the SR Ca^2+^ ATPase and the inositol 1,4,5-trisphosphate (IP3) receptor [143–146]. NSCs/NPCs are sensitive to regional changes in Ca^2+^ concentration. Bax-knockout SVZ NSCs are less prone to caspase-3 mediated cell death. Moreover, Bax is a major determinant of apoptosis in adult NSCs, and its action is mediated by the modification of IP3 receptor-mediated calcium flux [147]. Knockdown of either Bax or IP3 receptor significantly improved cell survival, thereby strengthening the role of ER in the PCD of NSCs/NPCs. In the adult neurogenic SVZ and SGZ, apoptotic NSCs are phagocytosed by unchallenged microglia [32].

#### The extrinsic apoptotic signaling pathway is non-functional in adult NSCs

Upon ischemic injury in the adult brain, neurons undergo extensive apoptotic cell death mediated by death receptor ligands, including TRAIL, Fas, and TNF-α [148]. Whether NSCs undergo apoptosis via the death receptor-mediated extrinsic apoptotic pathway in response to pro-inflammatory cytokines released by activated microglia is of great interest. In murine NSCs, activation of the Fas receptor by Fas ligand (FasL) does not induce apoptosis, suggesting that NPCs are insensitive to death receptor-mediated apoptosis despite the fact that the Fas receptor is endogenously expressed [149]. FasL treatment increased phosphorylation of ERK in a caspase-8 independent manner, which suggests that FasL promotes growth rather than death in NSCs [149, 150]. Similar to the effect of FasL on adult NSCs, TNF activation does not cause extensive apoptotic death in adult NSCs. In vitro TNF-α treatment for up to three days induced a low level of DNA fragmentation in adult SVZ NSCs, suggesting that adult NSCs have high thresholds for the induction of TNF-α induced activation of the extrinsic apoptotic pathway [151]. Sox-2 positive adult NSCs express TLR2 and TLR4, and when these cells are challenged with the TLR agonists, TNF-α has been found to be released from NSCs; however, this ligand did not promote either death receptor-engaged extrinsic apoptosis or the proliferation of adult NSCs [152].

The mechanism of protection against death-inducing ligands in adult NSCs has not been well characterized. In NSCs, FLIP, a well-established endogenous inhibitor of caspase-8, was found not to be responsible for the non-functional death receptor pathway [149, 150]. Instead, low expression of caspase-8 and the competitive binding of PED/PEA-15 to FADD altogether prevent caspase-8 processing in adult NSCs [153]. The resistance to death ligands is probably an intrinsic mechanism to conserve a pool of NSCs when cytokines are over-produced following/during CNS injury.

### Apoptosis of migrating neuroblasts

Elimination of erroneous or developmentally transient cell populations for error correction is one of the major functions of PCD during embryonic or early postnatal development. For example, a subset of erroneously migrating Purkinje cells are eliminated from their ectopic position in the cerebellum, a process which can be blocked by eliminating the Bax gene [154], suggesting that the elimination of cells that fail to migrate may be a common function of PCD to allow for error correction in the postnatal brain. Failure in neuroblast migration also appears to induce PCD in the adult brain. We observed that Bax-knockout mice exhibit a massive accumulation of ectopic neurons in the RMS where young neuroblasts are actively migrating [33]. Considering that dying cells are frequently observed in the migratory stream [155], it appears that some neuroblasts undergo PCD during their migration. In Bax-knockout mice—and due to the increased flow of neuroblasts through the relatively constant migratory tube generated by ensheathing astrocytes—glial cells were eventually displaced and the neuroblasts were released from the migratory tract. Interestingly, migrating neuroblasts express the N-Methyl-D-aspartic acid receptors (NMDARs) long before synaptic integration, and a single cell-level gene knockout of the NMDARs in neuroblasts resulted in PCD [156]. Considering that vesicular release of glutamate from the ensheathing astrocytes is a major source of NMDARs activation, loss of NMDA activation might contribute to the PCD of displaced neuroblasts. However, it should also be noted that migration-defective neuroblasts do not always undergo PCD, and a subset of cells appears to be differentiated into mature neurons [157]. Currently the impact of these ectopically survived neurons are unclear, but PCD of migrating neuroblasts may serve to prevent potential errors in neural “wiring.”

More recently, it was discovered that a subset of early neuroblasts (2–4 days after generation from transit-amplifying cells) in the DG also undergoes PCD [1]. Considering that neuroblasts at these periods in the SGZ begin to migrate toward the interior of the granular zone, it appears that PCD—as occurs during the neuroblast stage—is common in adult neurogenic regions. However, it is unclear whether PCD during this early phase of the neuroblast population is relevant to the elimination of erroneously migrating cell populations.

### Apoptosis of immature neurons during neuronal integration

It appears that the most extensive PCD occurs during the synaptogenesis of postmitotic neurons. In the normal adult brain, a considerable number of dying cells is readily observed using a variety of methods [155]. Most of these dying cells in the neurogenic regions are neuroblasts, and they can easily be identified by the expression of markers for immature neuroblasts, including DCX and PSA-NCAM. Lineage-tracing and birth-dating experiments also consistently suggest a 30–70 % loss of immature neurons during the formation of provisional synaptic contacts and subsequent synaptogenesis. For example, double labeling with apoptotic and neuronal markers has shown that PCD occurs during the maturation of newly generated neurons in the DG [158–160]. In addition, massive PCD is seen in the OB where young neuroblasts stop migrating and begin to differentiate [47, 50, 161]. PCD during the synaptogenic period is critical for the efficient establishment of synaptic connections. The mechanism that determines the survival of an optimal number of neurons during development is explained by the ‘neurotrophic hypothesis’ [5, 162]. The neurotrophic hypothesis assumes that the availability of neurotrophic signals released from target organs/neurons for the survival of neurons is limited. Under this condition, competition among the neurons for access to these trophic signals eventually determines how many cells are to remain. Identification of target-derived neurotrophic factors and microsurgery or gene perturbation experiments have consistently supported the neurotrophic hypothesis as a promising model for explaining developmental PCD. It seems likely that the mechanism of PCD in adult neurogenesis is also related to the neurotrophic hypothesis. For example, there is substantial evidence correlating conditions that increase neurotrophic signals, such as physical exercise or hippocampal learning, with reduced PCD of adult-produced neurons [86, 163–165]. By contrast, conditions that suppress the expression of neurotrophic signals, such as stresses or social isolation, enhance PCD. In this respect, at least, neurotrophic factors are involved in the survival of adult-produced neurons. Although it remains unclear what and how significant a competition for neurotrophic signals plays in the selection of surviving neurons, there is evidence demonstrating the presence of competition among newly produced neurons for survival. For example, the genetic elimination of the NMDARs in a subset of newly produced neurons using a single-cell knockout technique resulted in the reduced survival of the affected neurons, whereas total number of surviving neurons remained constant by increased survival of neighboring, unaffected normal neurons [166]. This result fits neurotrophic hypothesis since, losers (affected cells) will die more than winners (unaffected cells) under the selection pressure of limited amount of glutamate receptor activation or subsequent neurotrophic signals.

Competition for survival among immature neurons essentially occurs between homogeneously immature neurons, but immature neurons in the adult brain must also compete with pre-established mature neurons for new synaptic connections. Morphological tracing has shown that newly born neurons form synapses with mature neurons [50, 167–169], and they begin to receive inputs from afferent fibers by 2 weeks after birth [66, 170–172]. Considering that immature neurons efficiently incorporate into the neuronal circuit, they should be able to replace old connections. To accomplish such a replacement, either young neurons should have the ability to actively remove preexisting partners to form new connections, or old neurons should be spontaneously removed and provide room for young neurons. We have addressed this and related issues experimentally by blocking PCD in Bax-knockout mice. In Bax-knockout mice, PCD of DG neurons is completely blocked [73]. Accordingly, the number of DG neurons progressively increases with age in Bax-KO mice. In this situation, the outnumbered adult-produced, surviving neurons form synaptic connections with efferent and afferent neurons [34]. Considering that massive loss of young neurons is prevented by Bax deletion, and that surviving neurons successfully find their synaptic partners—which are relatively constant in number—the above observation suggests that young neurons can form synapses with actively eliminated preexisting neurons. In support of this, we have found that Bax-knockout mice showed relatively normal neural circuits formed by young neurons, but mature neuronal circuits were severely impaired, indicating that long-surviving adult-generated neurons can intercalate with mature neural circuits. In this respect, it appears that PCD of adult neurons is a means to maintain the integrity of neuronal circuits, and the failure of this process cannot be compensated by other biological events such as synaptic pruning, which occurs during embryonic development [173].

However, recent data have shown that conditional knockout of the Bax gene in the adult brain results in enhancement of pattern separation without affecting other major hippocampal functions [174]. This observation is somewhat contradictory to the neurotrophic hypothesis, because it implies that the prevention of immature neuronal death during the competitive period can enhance circuit efficacy. It is important to consider that the DG is involved in many different hippocampal functions in addition to pattern separation. In fact, it has been proposed that DG circuits mediated by immature neurons and mature neurons may have distinct functions. For instance, young neurons exhibit strong synaptic plasticity [172, 175] due to the lack of GABAergic inhibition [166, 175–177]. With this and other distinct cellular characteristics, immature DG neurons appear to mediate efficient pattern separation, whereas mature neurons mediate other functions such as sensory alignment [178]. Therefore, the enhancement of pattern separation following the prevention of PCD may be a transient event caused by an imbalance of immature vs. mature neuronal circuits. In fact, we also found that olfactory learning ability is improved in Bax-knockout mice while major olfactory sensation is virtually normal [33, 56]. Collectively, the functional impact of PCD in adult neurogenesis should be evaluated by examining the interplay between young neurons and mature neurons.

### Apoptosis of mature neurons already integrated in the neuronal circuits

Although the majority of neurons undergoing PCD in the adult brain are adult-generated immature neurons, mature neurons in adult neurogenic regions appear to undergo spontaneous PCD, which can cause the ‘renewal’ of old circuits by replacing dying mature neurons with newly produced ones. Birthdate tracing revealed that about 25 % of the DG neurons generated during a major developmental neurogenic period (postnatal day 6) in rats undergo PCD during the first 1–6 months after birth [72]. Adult-born neurons that survive beyond the PCD period appear to live at least 19–21 months in both rats and mice [49, 179]. On the other hand, newly produced OB neurons undergo PCD during the first 6–19 months after birth [50]. Less is known about the PCD of mature DG neurons in mice, but the total number of DG neurons is invariable in adulthood, indicating that PCD occurs in mature neurons in order to numerically match the adult-produced new neurons [73]. In this way, newly generated neurons appear to replace earlier born, older cells.

Although it is puzzling why new neurons should replace old neurons in limited spontaneous neurogenic regions, the presence of spontaneous cell replacement indicates that new neurons replacing damaged neurons in the diseased or damaged brain is possible.

### Autophagic cell death in adult neurogenesis

The majority of studies on PCD during adult neurogenesis to date are limited to apoptosis. Whether other types of PCD are operating during adult neurogenesis remains largely understudied. As the first report on non-apoptotic mode of PCD in adult NSCs, we reported that adult hippocampal neural stem (HCN) cells deprived of insulin committed to ACD despite an intact apoptosis pathway. Genetic suppression of Atg7, a component of the autophagy machinery, attenuated cell death, whereas treatment of the cells with Z-VAD-fmk, an apoptosis inhibitor, failed to attenuate cell death in such conditions [135]. Furthermore, the expression of autophagy marker proteins such as Beclin-1 and LC3 II were elevated; in stark contrast, Bcl-2 family proteins Bcl-2 and Bcl-X_L_ were down regulated [135]. To date, the underlying molecular mechanism of ACD has not been fully understood. Recently, we have reported that activation of GSK-3β, a well-known inducer of neuronal apoptosis, induced ACD instead of apoptosis following insulin deprivation [180]. Calpain is a cytosolic calcium-activated cysteine protease [181]. Calpain inactivation promoted ACD in insulin-deprived HCN cells, whereas calpain activation completely switched ACD to apoptosis, demonstrating that calpain mediates crosstalk between ACD and apoptosis in HCN cells [182]. Recently, the role of Beclin-1 and Ambra1-mediated autophagy has been also reported in adult neurogenesis at SVZ [183]. In adult neurogenesis, the mode of PCD in NSCs/NPCs may vary depending on the nurturing environment of the niche.

### Necrosis in adult neurogenesis

Necrotic death occurs rather rarely during physiological postnatal development in the brain. Under pathological conditions such as ischemic injury and inflammation, extracellular ATP released from nearby damaged cells or glial cells evokes necrotic death in recipient cells with purinergic receptors [184]. Generally, extracellular ATP triggers the apoptotic cascade in cells as an immediate consequence of the rapid rise in cytoplasmic Ca^2+^. However, at a higher degree of ATP insult, cells undergo necrosis instead of apoptosis [185].

Purinergic receptors exist in not only immune cells but also non-immune cells in the CNS. Adult NPCs also express the functional purinergic P2X7 receptor (P2X7R); therefore, stimulating receptor activation with extracellular ATP leads to necrotic death [186–188]. Furthermore, the purinergic receptor also mediates necrotic death in postmitotic cells. In dopaminergic neurons, extracellular ATP induced cell swelling and loss of integrity of the ER and plasma membrane—morphological hallmarks of necrotic death; these effects were blocked by treatment with a P2X7R antagonist [186].

Taken together, our understanding of the molecular mechanisms involved in necrotic death of adult NSCs/NPCs is limited due to a lack of evidence for necrosis during adult neurogenesis. Yet, the susceptibility of NSCs/NPCs to necrotic death under pathological conditions raises the possibility that adult neurogenesis may be perturbed through necrotic mode of PCD under certain conditions.

## Cellular regulation of PCD in adult neurogenesis

The stem cell pool is dynamically regulated by changes in the niche by extracellular molecules that are released from vascular endothelial cells, astrocytes, mature neurons, and peripheral sources [156, 189–194]. Humoral factors, neurotrophins, neurotransmitters, morphogens, and steroid hormones regulate the basal level of proliferation of NSCs and promote physiological survival of NSCs and newly produced neurons (Table 1). The regulatory roles of the factors in terms of survival of newly generated cells during adult neurogenesis is well-established in vivo studies; however, because the measures taken to date are limited to rate of volume growth, total numbers of TUNEL activity in BrdU-positive cells, caspase-3 activation, proliferation capacity, and differentiation potentials, the precise molecular mechanisms involved in PCD is not elucidated. Here, we review key regulators of neurogenic niche that have potential to control the intricate balance between survival and PCD in adult neurogenesis.

**Table 1:** The intrinsic and extrinsic factors for the control of adult neurogenesis

		Cell types			References
Factors		NSC	Neuroblast	Immature neuron	
Growth/Neurotrophic/Morphogenic factors	IGF-1	SGZ		DG	[53, 197–199]
	Insulin	SGZ			[135, 180, 182]
	NGF			DG	[204]
	BDNF			DG	[205, 206]
	Shh	SVZ			[228]
	Wnt	SGZ			[226, 227, 230]
Steroid hormones	Progesterone			DG	[236]
	Estrogen	SGZ			[231]
	Prolactin			OB	[234]
	Androgen	SGZ		DG	[232]
Neurotransmitters	Glutamate	OB, SVZ	SVZ	DG	[156, 166, 193, 211, 212]
	GABA		DG	OB, DG	[213–215]
	Serotonin	SGZ		DG	[216–218]
	Dopamine			DG, OB	[219, 220]
	Acetylcholine			DG, OB	[221–225]

Brain IGF-1 signaling is a major regulator of brain homeostasis, and accumulating evidence suggests that IGF-1 regulates adult neurogenesis via modulation of the number of newly generated neurons and synaptogenic outputs [53, 195–198]. Homozygous or heterozygous knock out of IGF-1 receptor results in increased number of apoptotic NSCs and retarded growth of several regions of the brain including the hippocampus, cerebral cortex, and cerebellum, respectively [199]. Neurotrophins, such as nerve growth factors (NGFs), brain-derived neurotropic factor (BDNF), and neurotrophin-3 (NT-3), are essential pleiotropic factors with survival and growth-promoting effects on selective neurons during embryonic development [200–203]. BDNF and NGF play a similar role as a pro-survival factor in the adult brain. It has been reported that continuous infusion of NGF for 6 days enhanced the survival of newborn DG neurons [204] and the reduced expression of BDNF or NT-3 (or their receptors) in mutant mice significantly reduced the survival of immature DG neurons [205]. Mechanistically, BDNF and NGF-mediated survival-promoting effect is directly mediated by their anti-apoptotic capacity; STS-induced PARP cleavage and caspase activation were suppressed by BDNF and NGF treatment to NSCs and the anti-apoptotic effect was mediated by activation of PI3K/Akt and MAPK pathway [206]. The multiple downstream events mediating the survival-promoting effects of neurotrophin on adult neurogenesis have begun to be elucidated [207].

Neurogenesis-related PCD are also controlled by various neurotransmitters. This is in sharp contrast to PCD occurring during development. In embryonic development, when significant levels of PCD are known to occur, the nervous system is still immature, and the influence of neurotransmitters is only marginal. Therefore, PCD during embryonic development is mainly controlled by neurotrophic signals, whereas neurotransmissions play important roles in the pruning of connections later. On the other hand, in the adult brain, NSCs and immature newborn neurons are all under the strong influence of mature neuronal circuits, and it appears that synaptic activity and neurotransmitters, either directly or indirectly, modulate the different stages of adult neurogenesis including PCD. Glutamate promotes survival by acting through NMDARs and metabotropic receptors (mGluRs) [208–210]. Genetic suppression of functional NMDARs have been shown to activate apoptosis in new neurons in the adult DG and OB, therefore suggesting that NMDARs activation-mediated control of the rate of cell death is maintained in the adult neurogenic niche [166, 211]. In the damaged brain following hypoxia/ischemia, NSCs are resistant to glutamate toxicity, and group II mGluR stimulation prevents cell death and increases the proliferation of type C cells by reducing cell cycle time in the SVZ [193], suggesting that glutamate also regulates NSC proliferation in this context via the activation of different receptors. SVZ-derived NPCs express functional mGlu1 and mGlu5 metabotropic glutamate receptors, and administration of mGluR1 antagonists (CPCCOEt) promotes the survival of NSCs, raising the possibility that endogenously activated mGluR1 can regulate natural cell loss by controlling the number of progenitors [212].

In the adult hippocampus, GABAergic synaptic input from parvalbumin-expressing interneurons promotes the survival of young DG neurons [213]. Suppression of GABA excitation in newborn neurons also impaired the survival of newborn neurons, a process mediated by the suppression of CREB activation [214]. Therefore, GABA-mediated depolarization and the subsequent initiation of CREB signaling appear to enhance the survival rate of newborn dentate granule cells [215].

Other neurotransmitters serotonin (5-HT) [216–218], dopamine (DA) [219, 220], and acetylcholine [221] have survival-promoting activity during adult neurogenesis predominantly via 5-HT1A receptor subtype, D1-like receptor, and α7-containing nicotinic acetylcholine receptors, respectively [222–225].

Morphogens sonic hedgehog (Shh) and Wnt are responsible for the maintenance of the stem cell population in the adult brain [226, 227]. A loss-of-function study using transgenic *Shh*^*n/c*^*;N*^*cre*^ mice showed that disturbance of Shh signaling reduced the number of neurospheres formed and the mechanism of action is at least partly via regulation of PCD in the neurogenic region [228]. Impaired Wnt/β-catenin signaling activates caspase-3 in NSCs of DG [229]. Mature dentate granule neurons in SGZ is a source of secreted molecule that regulate activation of quiescent NSCs via secreted frizzled-related protein 3 (sFRP3), which is a naturally secreted inhibitor of Wnt; loss-of-function study using adult *nestin-CreER*^*T2*^*::Z/EG*^*f/*+^::*sfrp3*^−/−^ mice has shown profound increase in postmitotic cells compared to wild-type mice [230]. In addition to the above mitogenic factors, steroid hormones progesterone, estrogen [231], and androgen [232] also control adult neurogenesis. Progesterone in particular enhances neuronal survival by receptor-mediated signaling [233–236].

Clearly, dynamic changes in the levels of the various cellular factors will serve as the potential determinant for the cell death rates of NSCs, thereby the extent of neurogenesis in the adult brain. However, the detailed mechanisms by which each factor affects the PCD of NSCs are lacking in majority of the studies and there is a big gap in our understanding between the physiological outcomes of the cellular factors and their action mechanisms in regulation of adult neurogenesis.

## Systemic regulation of PCD in adult neurogenesis

Considering that one of the major roles of the nervous system is to sense external information and to decode the information for behavioral/functional outcomes, it is no wonder that adult neurogenesis and the resulting brain plasticity is intimately associated with the external sensing and behavioral responses of an organism at the systemic level. It has been proposed that the proliferation of NSCs requires long-term lag periods to have a functional impact on brain function, because newly produced neurons require 3–6 weeks for system integration [237]. On the other hand, PCD mainly occurs 2–4 weeks after birth, indicating that regulation of PCD can immediately regulate neurogenesis-related brain plasticity. In this respect, it is tempting to speculate that the control of NSC proliferation and PCD is somewhat suitable to maneuver various changes at the systemic level, such as experience-based learning and stress, nutritional condition, and aging [86, 238].

### Experience

It has been reported that environmental stimuli or the experience of the animal affect the extent of neurogenesis. For instance, an enriched environment or physical exercise enhances the survival of newly generated hippocampal neurons [165, 208, 239–241]. Enrichment of the odorant environment at a critical period also promotes the survival of OB neurons in mice [179, 242, 243]. On the other hand, odor deprivation by naris closure reduced neurogenesis in olfactory bulb and increased the death of the adult-generated neurons [244]. Hippocampal learning such as eye blink conditioning, a water maze task, or olfactory learning such as odor discrimination promote the survival of newly generated neurons in the DG and OB, respectively [164, 245, 246]. On the other hand, aversive experience such as stress or social defeat reduced adult neurogenesis [247, 248]. The induction of long-term potentiation, which is the cellular basis of learning and memory, also promotes the proliferation and survival of new neurons in the DG of rats [249, 250], suggesting that the use of neural circuits may promote the survival of new neurons. It remains unclear how neural activity ultimately controls PCD, although humoral factors, hormones, and neurotransmitters all appear to contribute to the regulation of PCD either directly or indirectly [49, 205, 251–253]. Recently, molecular links between experience-based learning and adult neurogenesis have been reported. Running, an element of environmental enrichment, increased DG neurogenesis and BDNF levels [254]. Running also induced the uptake of blood IGF-1 into the brain and peripheral infusion of anti-IGF-1 blocked running-induced increases in hippocampal neurogenesis [255]. During the development of newborn interneurons in olfactory bulb, sensory experience is a critical factor and it regulates dendritic branching and spine formation by 5 T4 oncofetal trophoblast glycoprotein and the neuronal Per/Arnt/Sim domain protein 4 transcription factor [256].

### Energy metabolism

Adult neurogenesis is dependent on numerous stimulating and inhibiting extrinsic factors, including dietary components. There is growing evidence suggesting that neurogenesis is impaired by high-fat diet-induced and genetic forms of obesity. The excessive intake of carbohydrates and saturated fatty acids leads to obesity and alterations in neurogenesis [257]. Indeed, the link between neuropathology and obesity has been shown by demonstrating that weight reduction improves cognitive function [258].

In recent years, factors that may mediate the correlation between diet composition, obesity, and adult neurogenesis have been investigated. Among these factors, appetite-related mediators, including circulating hormones such as leptin, ghrelin, and the pro-inflammatory cytokine TNF-α are being examined to characterize their potential role in mediating neurogenic responses to macronutrients. In vitro and in vivo studies have demonstrated that leptin and ghrelin stimulate adult neurogenesis by activating receptors on newly generated neurons in the hippocampus, hypothalamus, and brainstem regions [259–261]. In addition, it has been shown that hippocampal neurogenesis is impaired in rodent models of diabetes [262]. Diabetes impairs hippocampal function through glucocorticoid-mediated effects on both new and mature neurons, and the impairment is reversed when elevated levels of circulating corticosterone are normalized. Neurogenesis in the SVZ appears to be mediated by insulin, glucocorticoids, and BDNF [263].

Neuroplasticity changes including neuronal activation, neurochemical phenotype, synaptic connections, and dendritic growth in the hypothalamus can be stimulated by dietary factors even in adulthood. This hypothalamic neurogenesis is proposed as a compensatory mechanism that regulates energy balance by replacing dead neurons [103]. Several factors, including a variety of metabolic signals known to change energy supply via nutrient-sensing and satiety, may be involved in hypothalamic neurogenesis.

### Aging

It is well known that the extent of adult neurogenesis is significantly associated with age, and the numbers of NSCs in most adult neurogenic regions are reduced with age [264]. For example, the proliferation of NSCs or neuroblasts in the rodent SVZ is decreased by 50–70 % at 10–14 months of age [265–267], resulting in a >80 % decrease in the final number of newly produced neurons in the OB [266, 268]. Similarly, decreases in NSC proliferation and adult neurogenesis are also observed in the DG of 13 month old rodents [241]. On the other hand, the reduction in neurogenesis mainly occurs during 3–6 months of age in the SCZ [269], suggesting that the time-course of age-related changes in adult neurogenesis differs depending on the region of the brain.

Although it is clear that the extent of adult neurogenesis declines with age, this spontaneous reduction in NSC number is theoretically caused by multiple events, including PCD of precursors, reduced mitotic potential, and terminal differentiation. It appears that neural progenitors terminally differentiate into astrocytic cells in the DG [270]. In addition, age-related changes in the proliferating potential of NSCs (i.e., the transition from an active to a quiescent state) is also observed in the DG [270]; these are mediated by changes in the expression of cell cycle-related genes in NSCs [271, 272]. However, the contribution of PCD to NSC loss remains unclear, mainly due to technical difficulties in investigating this issue. In addition, adult neurogenic regions are in fact complex, and proliferating cells and differentiated cells are not well segregated spatially. As molecular markers for cell type identification are often downregulated during the early phase of PCD, the specific cell types that are actually degenerating in vivo are often not easily determined.

## Conclusions

In contrast to the great deal of interest in neuronal cell death during the development and pathogenesis of neurological disorders in the CNS, relatively little is known about the molecular mechanisms of PCD underlying adult neurogenesis. Adult neurogenesis continues throughout life. However, the number of mature neurons that become functional is several orders less than the number of proliferating neural precursors. Furthermore, in spite of continuous neurogenesis, the volumes of the SVZ and DG remain constant during adulthood. This means that there are cellular mechanisms working to eliminate the substantial fraction of proliferating or newborn cells and maintain the appropriate cell number in neurogenic regions.

Several critical questions need to be answered to understand the biological principles regulating this intricate balance between neurogenesis and the maintenance of total cell number in neurogenic regions. One key question is what molecular mechanism(s) is/are responsible for turnover of NSCs during adult neurogenesis. Histological and stereological studies in several genetic animal models targeting cell death molecules have suggested that the apoptotic pathways involved in neurogenesis are just beginning to be identified. However, more detailed knowledge of the molecular pathways regulating PCD of NSCs is needed for improved therapeutic design aimed at stem cell therapy. In addition, lack of knowledge of the complex interrelation between apoptosis and other non-apoptotic cell death mechanisms could be a hurdle to harnessing the possible therapeutic potential in adult neurogenesis.

Second, it remains unknown how neurogenesis is linked to the PCD-mediated elimination of “extra” new neurons. It is interesting to hypothesize that NSCs incapable of going through the destined developmental program would be doomed to removal by PCD. If this is the case, then what could the molecular trigger be for the initiation of PCD in such NSCs? Do we need a theoretical model, similar to the “neurotrophic theory”, to explain the molecular events behind PCD in adult neurogenesis?

Whether differences exist in molecular mechanisms of PCD between embryonic and adult neurogenesis stages or between basal on-going PCD and PCD associated with pathological conditions during adult neurogenesis are intriguing questions. Another key question is whether each developmental stage of neurogenesis will require distinct cell death machinery and regulatory programs. For example, it is not known whether quiescent NSCs (e.g., type B progenitors in the SVG) undergo PCD. If so, it has never been asked whether there are mechanistic differences between cell death in quiescent NSCs and in the proliferating populations of NSCs. The developmental stage at which cell death occurs, and whether cells may have different propensities to undergo specific PCD programs depending on their developmental stage, will be very challenging to elucidate. To address these questions, we will need extensive comparative analyses of cell death at each developmental stage of the entire process of neurogenesis.

Finally, clinical application would be feasible if we can further elucidate the mechanism of PCD during adult neurogenesis. Because many studies have not directly measured the PCD of endogenous cells in vivo upon transplantation, potential hazards or benefits cannot be fully predicted. An understanding of the molecular mechanism underlying PCD during adult neurogenesis, which will contribute to enhancing the survival and differentiation of endogenous NSCs, will advance the clinical applications of NSC therapy.

## Ethics approval

Ethics approval and consent to participate, consent for publication, and availability of data and material are not applicable to this study.

## Abbreviations

5-HT: Serotonin
ACD: Autophagic cell death
BDNF: Brain-derived neurotropic factor
BrdU: Bromodeoxyuridine
Carbon-14: C^14^
CNS: Central nervous system
DA: Dopamine
DCX: Doublecortin
DISC: Death-inducing signaling complex
ER: Endoplamic reticulum
FasL: Fas ligand
GABA: Gamma aminobutyric acid
HCN: Hippocampal neural stem
HGF: Hepatocyte growth factor
mGluRs: metabotropic glutamate receptors
NCAM: Neural cell adhesion molecule
NG2: Neural/glia antigen 2
NGFs: Nerve growth factors
NMDARs: N-Methyl-D-aspartic acid receptors
NPCs: Neural progenitor cells
NSCs: Neural stem cells
NT-3: Neurotrophin-3
OB: Olfactory bulb
P2X7R: Purinergic P2X7 receptor
PCD: Programmed cell death
RGCs: Retinal ganglion cells
RIP: Receptor-interacting protein
SCZ: Subcallosal zone
sFRP3: secreted frizzled-related protein 3
SGZ: Subgranular zone
Shh: Sonic hedgehog
SVZ: Subventricular zone
TNF: Tumor necrosis factor
TRAIL: TNF-related apoptosis-inducing ligand
